# Validation of empirical equations for determining the suitability of the territory for the setting of cemeteries – Implications for worldwide application

**DOI:** 10.1016/j.heliyon.2024.e40639

**Published:** 2024-11-26

**Authors:** Miguel Falconí-Vélez, Tania Crisanto-Perrazo, Wendy Cachaguay Viracucha, Silvana Suntaxi Pachacama, Jonathan Guayasamín-Vergara, Arianna García-Bazurto, Theofilos Toulkeridis

**Affiliations:** a Departamento de Ciencias de la Tierra y la Construcción, Universidad de las Fuerzas Armadas, Av. General Rumiñahui S/N y Ambato, 171103, Sangolquí, Ecuador; b Facultad de Ciencias Básicas, Universidad Técnica de Manabí, Avenida José María Urbina, Portoviejo, EC130105, Manabí, Ecuador; c Facultad de Ingeniería Civil y Ambiental, Escuela Politécnica Nacional, Av. Ladrón de Guevara E11-253, 170525, Quito, Ecuador; d Universidad Internacional Iberoamérica de México, Calle 15 num. 36, entre 10 y 12, CP 24560, Campeche, Mexico; e Investigadora Independiente, Avenida Uruguay, Portoviejo, EC130116, Manabí, Ecuador; f School of Geology, Aristotle University of Thessaloniki, 54124, Thessaloniki, Greece

**Keywords:** Environment equations, Environment suitability, Environmental pollution, Territorial planning, Land use

## Abstract

The final disposal of corpses is one of the activities that generates the greatest pollution. Chemicals, embalming fluids, greenhouse gas emissions, and other factors trigger contamination of soils and water sources in towns near cemeteries. This study aimed to validate empirical environmental equations to determine the suitability of territories for cemetery location in several cantons of Central Ecuador, address study variables measured in the field, and update the bibliography as a rapid decision-making tool for decentralized governments. To achieve this objective, we used various determined variables, such as the phreatic level and slope in the field, and evaluated other variables and values, including the distance to water sources, precipitation, soil type, cemetery age, temperature, number of graves, geological faults, and population density, for 15 cemeteries. The resulting values were used in 135 equations to determine the territorial suitability of the cemetery locations. The outcomes allowed the equations to be segmented into three groups: those that coincided with the initial theoretical category assigned to the reference studies; those that had a higher or lower category than the original studies; and those that did not coincide with the original studies. Despite their limitations, the equations developed in this study may provide a fast and inexpensive solution to define the construction setting of cemeteries worldwide. Furthermore, the results can be used for norms and standards in local territorial planning plans while using strict initial categorization controls.

## 1 Introduction

Owing to persistent population growth and territorial expansion of cities, interest in environmental concerns related to the final disposal of corpses and cemetery location has been increasing [[1], [2], [3]]. This topic has become critical in cases of excess mortality [[4], [5], [6]], such as the severe acute respiratory syndrome coronavirus 2 (SARS-CoV-2), also known as COVID-19 [7,8], which has resulted in 6,923,628 fatalities from 2019 to 2023 [9]. Most corpses are buried in cemeteries [10,11]. However, cemeteries may become a means of direct soil [2,[12], [13], [14], [15], [16]] and water body contamination because of leachate infiltration [[17], [18], [19], [20]]. Gonçalves V. et al. [18] found high bacteriological and physicochemical levels in cemeteries, higher than those of water from other sampling points [21]. Notably, the toxicity of these leachates depends on the presence of organic compounds [22].

In the case of Latin America, in Puno, Peru, 71 environmental impacts were identified in the areas surrounding a cemetery in Paucarcolla District [23]. In the physical environment, 35 of the 71 impacts (49.30 %) affected the soil, air, and water because of putrescine, cadaverine, pathogenic microorganisms, heavy metals, radioactive isotopes, and dioxins, which can migrate from the soil to the phreatic levels underground, compromising the quality of the environment [2,14,20]. In the biological environment, five impacts (equivalent to 7.04 %) have been observed, and in the socioeconomic environment, 31 impacts (43.66 %) have been observed, such as impacts on the landscape, perception of environmental impacts, and local commerce [20]. Further research was conducted on existing environmental problems in the cemeteries of Pichincha Province, Central Ecuador [12].

A corpse management plan [[24], [25], [26]] can prevent the spread of infectious diseases and protect public health [1,11,24,27]. Thus, the Ministry of Public Health of Ecuador (MSP) issued Ministerial Agreement (AM) 3523 in 2013, which established guidelines for optimal conditions for cemetery location, and AM 192 in 2018 as regulations for managing corpses [28,29].

Cadaveric decomposition necessitates the evaluation of environmental and geographical characteristics of current and future locations of cemeteries [18,30,31]. Therefore, a methodology was created to evaluate the suitability of a territory for the location of cemeteries by employing empirical equations [12] obtained using an analytical hierarchy process of variables (AHP). The equations have been derived using a hierarchical process employing the Saaty matrix [[32], [33], [34], [35]]. Using the Saaty matrix, various levels of influence were defined for each variable, and relative values were assigned to cover all possibilities quantitatively [12]. Subsequently, nine equations containing 10 variables (water table, distance to water sources, precipitation, slope, soil type, cemetery age, temperature, number of graves, geological fault, and population density) were established and evaluated theoretically.

Pollution from cemeteries is an emerging environmental issue from the community to the global level. Zychowski and Bryndal [20], and Cai et al. [1] demonstrated that pollution from cemeteries influences the environment. Globally, this problem has resulted in a high level of contamination in water and soil sources because of population growth and poorly situated cemetery locations [36].

Therefore, the primary aim of this study was to conduct a field validation of the established empirical environmental index equations to determine the suitability of the territory for the cemetery location in Central Ecuador. Additionally, this study examined many variables (groundwater level, distance to water sources, precipitation, slope, soil type, age of the cemetery, temperature, number of graves, geological fault, and population density) by using Saaty's prioritization matrix. The main contribution to the literature is a rapid decision-making tool for decentralized governments that facilitates cemetery construction and minimizes the probability of water and soil contamination.

### 1.1 Corpses as a contamination factor

Approximately 99 % of the living human body mass consists of six essential chemical elements: oxygen (65 %), carbon (18 %), hydrogen (10 %), nitrogen (3 %), calcium (2 %), and phosphorus (1 %) [12,37]. The remaining percentage is composed of various elements, primarily K, S, Na, Cl, Mg, I, Fe, and Zn [38]. Metabolic processes, which involve the combined action of lipids, carbohydrates, proteins, amino acids, and other internal factors (such as weight, age, and sex), contribute to the balance of all systems of a living organism [3,11,39,40]. After death, an adult human weighing 70 kg releases up to 40 L of leachate, which contains 60 % water, 30 % mineral salts, and 10 % complex nitrogenous substances (such as putrescine and cadaverine) [11,12,41]. Therefore, viewing the deceased as waste that must be managed appropriately is necessary, while respecting regional ethical principles [26].

Burial practices vary widely among cultures and, in many cases, burial sites have been established. According to previous studies, signs of decomposition can persist for centuries or millennia after burial, because substances may be released that alter the chemical composition of the soil, affect groundwater, and become sources of dangerous infectious diseases [13].

The decomposition of a corpse, a process that develops sequentially in stages [42,43], can be influenced by various factors, such as climate, temperature, pH, burial depth, and the presence of scavenging insects, that may accelerate or slow its natural progress [1,3,43]. The decomposition of a corpse in soil exhibits a sigmoidal pattern [18,44,45]. Forensic biology subdivides the decomposition of a corpse into five stages of variable duration [[45], [46], [47]]. The first stage is called “fresh,” it begins at the time of death and culminates with the initial detection of an obvious odor, marking the beginning of internal decomposition caused by bacterial activity. During the second phase, “swelling or putrefaction,” the body swells owing to the accumulation of gases produced internally by the metabolic activity of anaerobic bacteria [48,49]. The third stage is called “active decay,” during which the following occur: butyric fermentation and black rot, the collapse of the body as gases are released, and decomposition that results in an odor that becomes particularly strong [44,50]. The fourth stage, called “advanced decay,” involves butyric/ammoniacal fermentation. During the fourth stage, the body becomes dehydrated, initially retaining some fleshy tissue and then emitting a cheese-like odor, and its ventral surface becomes moldy owing to fermentation [48,51]. Lastly, in the fifth stage, called “dry decay,” the body is largely desiccated and is mainly composed of bones and hair. During the fifth stage, the decomposition rate is slow, and the predominant odor is similar to that of common dirt and garbage [45,49,50]. Under ideal conditions, the decomposition process is completed within the typical resting period, which varies between 15 and 25 years, leading to the complete skeletonization of the corpse [48].

A study conducted in a cemetery in Bogotá, Columbia, determined that water samples near graves harbored many microorganisms and indicators of contamination, including *Salmonella* choleraesuis, *Enterobacter* cloacae, *Clostridium* spp., total coliforms, and fecal matter [52,53]. Furthermore, in three cemeteries in Carazinho, Brazil, high levels of contamination by heavy metals such as Cu, Pb, Zn, Cr, Fe, and Mn have been detected [18,[54], [55], [56], [57]]. These contaminants [58] pose potential risks to human health, especially heavy metal contamination of the water table [2,13,16,19,20,59].

In a cemetery in South Africa, there were where high concentrations of metals, for example, B (5.99 mg/kg), Mn (430.66 mg/kg), Ni (440.63 mg/kg), Zn (7.76 mg/kg), Cu (17.39 mg/kg), and As (0.39 mg/kg). Because of current and past burial practices, such concentrations can accumulate deep in the soil of cemeteries [10,42,[60], [61], [62]].

In forensics, the surrounding environment is described as an “island of cadaveric decomposition” (ICD) [18,56]. ICDs release various chemical compounds, including gases (H_2_S and NH_3_), volatile organic compounds, and N [55,57]. The main contaminants of ICDs are elements used in the manufacture of coffins, such as wood, metal, sealants, and varnishes, because they can release toxic or polluting chemicals into the environment as they degrade or corrode [61,63]. These contaminants can potentially be released into soil or water, which has a negative environmental impact. For example, chemicals used in varnishes and sealants in wooden coffins may contain compounds that are harmful to the environment. Similarly, the corrosion of metal coffins can release heavy metals and other toxic compounds [15,46,57,61,64].

Because of the decomposition process, cemeteries are sources of microorganism proliferation and can increase the concentrations of metals and organic compounds in the environment [1,43,52,59], which can infiltrate the water table and subsequently contaminate soil and nearby water sources [13,16,19,20,59]. Notably, studies have demonstrated that unsaturated soils stop the passage of rainwater into gravel [21,59,[65], [66], [67], [68]].

AM 3523 states that cemeteries must be located on land with dry soil because it acts as a barrier that stops the flow of rainwater toward graves, thereby preventing the transport of microorganisms to groundwater sources [21,[65], [66], [67]]. Previous studies [12,69] have emphasized the importance of studying factors related to humidity. This could be due to precipitation in the area and the slope, as microorganisms at the hydrological level are directly influenced by the presence of a greater contribution of water in the environment, thereby accelerating or decelerating the decomposition process of corpses [70,71]. Furthermore, the putrefaction process is driven by high temperatures; thus, the range of favorable temperatures is 21–38 °C [69]. The slope and existence of nearby geological faults also affect the loss of soil nutrients because global faults cause erosion [67,72]. Therefore, in areas with relatively steep slopes, the released leachate tends to move toward sections of the sub-basins, and in the case of geological faults, the situation worsens because they facilitate the dispersion of pollutants in the environment. The water table is a sensitive variable for the location of a cemetery because it helps prevent contamination of the soil and nearby water sources, whether underground or at the surface [13,20,59]. AM 3523 [29] states that the water table should be at least 2.5 m deep and located at a minimum distance of 200 m from sources of drinking water (e.g., springs, springs that feed city supply wells, rivers, and irrigation canals), with the aim of preserving water bodies.

The age of the cemetery and the number of graves is the final relevant parameters [12] because of their direct relationship with the decomposition of the corpse and, therefore, the production of leachate. Similarly, the MSP established that the intervention or influence of human activity should be minimal in sectors surrounding the cemeteries [29].

## 2 Materials and methods

This study was based on 70 cemeteries evaluated based on field, bibliographic, and other studies [63]. Subsequently, the Saaty prioritization matrix was applied. Finally, 13 cemeteries were used and distributed within the five categories determined in the literature: not suitable, not very suitable, moderately suitable, very suitable, and totally suitable. Finally, Eqs. (1), (2), (3), (4), (5), (6), (7), (8), (9)) [12] were validated using international standards, an updated bibliography, the free version of the geolocation software ArcGIS 10 Pro, and field measurements.

To theoretically validate Eqs. (1), (2), (3), (4), (5), (6), (7), (8), (9)) in other latitudes, we analyzed cemeteries, such as Asia Cañete in Peru, Shenzhen Longshan in China, Palmira in Colombia, and Nuestro Padre Jesús de Murcia in Spain. Values for variables, such as distance to water sources and slopes, were established using Digital Elevation Models available in Earth science data collections based on NASA Earthdata [73]. Soil types were obtained using the FAO Soil Portal and UNESCO World Soil Map [74]. The distance to geological faults was obtained using maps developed by Carlotto et al. [75], Gomez-Novell et al. [76], and Wang et al. [77]. Data on cemetery age, temperature, number of graves, and population density were obtained from the official pages of the districts, municipalities, and states studied. The maximum annual precipitation was obtained from the precipitation maps and previous reports by Diaz et al. [78], Doswell et al. [79], Liu et al. [80], and the Directorate of Meteorology and Atmospheric Environmental Assessment (SENAMHI) [81]. The primary variable was the water table in each cemetery, which was obtained from previous studies by Acosta et al. [82], Lancia et al. [83], Tomas et al. [84], and Cooper et al. [85]. The equations were validated according to Crisanto-Perrazo et al. [12].

For Eqs. (1), (2), (3), (4), (5), (6), (7), (8), (9)), the variables were measured in the field during the dry season and were derived from updated bibliographic sources.

**10 variables**

(1) X=0.2951A+0.2126B+0.1498C+0.1036D+0.0698E+0.0452F+0.0452G+0.0292H+0.0292I+0.0203J

**9 variables**

(2) X=0.3119A+0.2206B+0.1524C+0.1033D+0.0683E+0.0436F+0.0436G+0.0282H+0.0282I

**8 variables**

(3) X=0.3311A+0.2394B+0.1551C+0.1030D+0.0672E+0.0427F+0.0427G+0.0288H

**7 variables**

(4) X=0.3543A+0.2392B+0.1573C+0.1017D+0.0650E+0.0413F+0.0413G

**6 variables**

(5) X=0.3825A+0.2504B+0.1596C+0.1006D+0.0641E+0.0428F

**5 variables**

(6) X=0.4185A+0.2625B+0.1599C+0.0973D+0.0618E

**4 variables**

(7) X=0.4673A+0.2772B+0.1601C+0.0954D

**3 variables**

(8) X=0.5396A+0.2970B+0.1634C

**2 variables**

(9) X=0.6667A+0.3333B

where:

A = Groundwater levels, B = Distance to water sources, C= Precipitation, D = Slope E = Type of soil, F= Age of the cemetery, G = Temperature, H= Number of graves, I= Geological Fault, J = Density Population.

For the validation of equations Eq (1) to Eq (9), the variables were measured in the field in the dry season and from updated bibliographic sources.

### 2.1 Study area

**Fig. 1:**
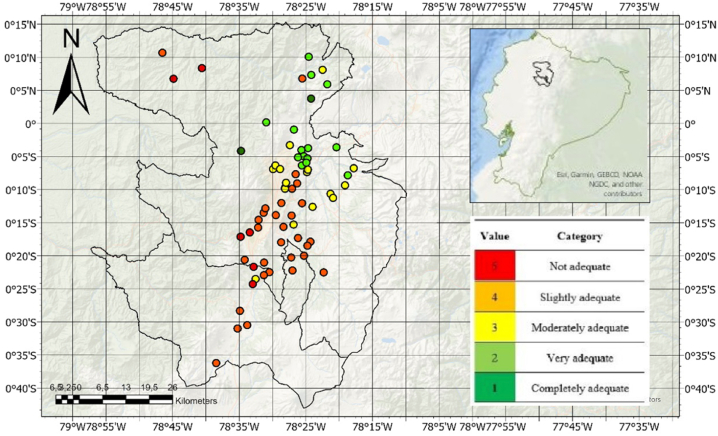
Study area in Central Ecuador. The map indicates the study points of the 70 cemeteries classified into five study categories.

The study area covered the cantons of Mejía, Quito, and Rumiñahui, Pichincha Province, in Central Ecuador. The study area is located at altitudes ranging between 2550 and 2886 m above sea level within the Inter-Andean Valley and is surrounded by various active and extinct volcanoes [[86], [87], [88]]. Cemetery selection was conducted using criteria from the literature [12] and according to the categories to which the cemeteries belonged (Fig. 1).

### 2.2 Sample collection

The 70 cemeteries were classified into five categories, from which a representative sample was obtained per category. Three cemeteries per segment were selected, because a statistical trend was observed using this number. Subsequently, the analytic hierarchy process was applied using the Saaty matrix and the weighted linear sum, with the objective of selecting the cemeteries to sample [89]. For the 14 selected cemeteries, visits by residents of nearby towns were conducted to verify the accessibility, security, and entry permit criteria. Subsequently, Ayapamba La Capilla cemetery was excluded. Thus, the final selected cemeteries were as follows: Lumbisí, La Libertad de Chillogallo, Tambillo, Aloasí, Nanegal, Descanso siempre, Yaruquí, Puéllaro, Chavezpamba, Uyumbicho, Guangopolo, Nono, and Tababela and the two cemeteries [12], Tumbaco and Calderón, used for the theoretical validation of the empirical equations.

### 2.3 Study variables

The variables analyzed and measured in this study were as follows: depth to the phreatic level, distance to water sources, precipitation and temperature, slope, soil type, cemetery age, number of graves, geological faults, and population density. For the water tables, values were obtained in the field using WDJD-4 multifunction digital DC resistivity/IP meter model equipment, using the 4P-VES method (Schlumberger). This method yielded a series of graphs based on the groundwater levels of each sample cemetery, obtained using EarthImager 1D software (Version 2.0.5) and 1D resistivity inversion software (Nwankwo & Emujakporue, 2020). The distance to water sources was obtained using geographic information systems, such as ArcGIS and Google Earth. The distance between the cemetery and nearest water source was measured linearly. Precipitation and temperature data were obtained from the National Institute of Meteorology and Hydrology in Ecuador (INAMHI) for 10 years, from 2003 to 2013 [90]. Ecuador is a mountainous area with considerable slopes in each of its cantons [88]. Using an AL03 clinometer, the values of degrees of inclination were obtained, which were transformed into values of the percentage of slope as a variable in the equations to be validated. The soil type was determined using sieving soil granulometric analysis performed in the soil laboratory of the University of the Armed Forces ESPE, using the ASTM D422 standard [91]. Once the values were obtained, the textural triangle method based on the United States Department of Agriculture (USDA) system was used to determine the textural class of each of the soil profiles [92]. Data on cemetery ages and number of tombs were acquired via field visits and bibliographic information available on the web and from previous literature reports [12]. The location of geological faults was obtained using the Geographic Information System (ArcGIS), cartography on the Geoportal website of the Geological and Energy Research Institute from Ecuador (IIGE) called “Map of Geological Faults,” and other sources [93]. Finally, the population density was obtained from the statistical data of the 2023 census conducted by the National Institute of Statistics and Censuses from Ecuador (INEC) [5].

### 2.4 Validation of empirical equations for determining suitability of territory in setting of cemeteries

To validate the empirical equations (Eqs. (1), (2), (3), (4), (5), (6), (7), (8), (9))), we created a spreadsheet that implemented these equations, generating segmentation values ranging from 1 to 5. These values were then used to evaluate the level of suitability of the studied cemetery land. Based on the established criteria, a cemetery may be evaluated as critical or unsuitable if, when adding the data of its variables, the result of applying the developed equations ranges between 4.01 and 5.00, thereby presenting a greater probability of environmental contamination. Conversely, the other categories were slightly adequate (range, 3.01–4.00), moderately adequate (range, 2.01–3.00) very suitable (range, 1.01–2.00), and completely adequate (4.01–5.00). An adequate oscillatory range was 0.01–1.00 [12].

## 3 Results

**Fig. 2:**
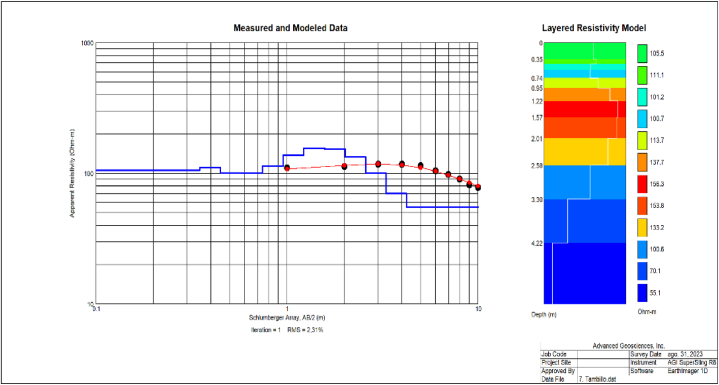
Resistance versus depth of the Tambillo, and the inversion point of this cemetery.

The resistivimeter operates on the principle of soil resistivity, characterized by the soil's ability to oppose electric current flow [94]. In situ, a 4P-VES array (Schlumberger) was used to determine the water table in each of the fifteen sampled cemeteries. Fig. 2, for instance, illustrates the work conducted in the Tambillo cemetery, where black dots indicate in situ measurement points. The blue line represents a resistance model linked to the scale, and the chromatic symbol on the right shows the relationship between model values and water table depth. The water table is identified as the lowest resistance point, referred to as the “inversion point,” due to the characteristic inverted bell-shaped curve [94].

In Tambillo, the lowest inversion point was approximately 55.10 Ohm-m. This corresponds to a depth of 4.22 m, as indicated by the scale on the right side of the graph, complying with AM 3523 regulations [29], where the minimum burial depth of human bodies is 2.50 m. According to the recent INEC census, Tambillo has a population density of 222 inhabitants/km^2^, and its cemetery was established in 1950; it currently has approximately 2,000 graves [5]. The closest body of water is located approximately 586.46 m from the cemetery, complying with Article 16 of AM 3523 [29]. In the analysis of precipitation, data from a 10-year period obtained from the nearest meteorological station showed maximum precipitation in 2008, with approximately 2032.30 mm/year. The soil exhibits characteristics that define it as clayey, with a distance from the geological fault of 10 m and a slope of approximately 10 % [90]. The information collected in the field and/or through the aforementioned highly reliable bibliographic sources of the remaining cemeteries is presented in Table 1.

**Table 1:** Values of variables studied in the sample. The letters correspond to the variables in Eqs. (1), (2), (3), (4), (5), (6), (7), (8), (9)).

Cemeteries	Variables									
	A	B	C	D	E	F	G	H	I	J
Lumbisí	2,18	701	1897	3	sandy clay loam	1970	17	200	1	1083
La Libertad	2,95	402	2241	22	sandy clay loam	1994	7	130	2	191
Tambillo	4,33	586	2032	10	clayey	1950	14	2000	0,01	222
Aloasí	4,91	188	1383	1	sandy clay loam	1950	10	1000	0,3	196
Nanegal	3,83	147	3042	15	sandy clay loam	1970	20	500	2	6
Descanso Eterno	4,11	116	1532	15	sandy clay loam	1980	17	400	0,12	3802
Yaruquí	4,18	625	1189	5	sandy clay loam	1985	14	3500	1	276
Puellaro	0	1663	907	16	sandy loam	1910	17	1000	0,11	19
Chavezpamba	4,03	1479	1900	9,38	sandy clay loam	1910	17	300	2,8	47
Uyumbicho	2,47	1119	1981	1,28	sandy clay loam	1940	14	1000	0,53	294
Guangopolo	4,52	495	1897	13,1	sandy clay loam	1950	17	470	0,76	422
Nono	4,19	31	1360	4,83	Loamy sand	1909	10	710	0,61	14
Tababela	2,77	218	1180	8,53	sandy clay loam	1952	14	600	2	152
Tumbaco	3,79	620	1180	3,06	sandy loam	1979	17	5100	1,95	1209
Calderón	3,73	1615	1532	3,44	sandy clay loam	1934	17	800	1,27	3184

***Note.*** A = Groundwater levels, B = Distance to water sources, C= Precipitation, D = Slope E = Type of soil, F= Age of the cemetery, G = Temperature, H= Number of graves, I= Geological Fault, J = Density Population.

To validate the method, after presenting the data for the variables considered in the equation solutions and utilizing a previously programmed spreadsheet, the respective equations (Eqs. (1), (2), (3), (4), (5), (6), (7), (8), (9))) were executed. This procedure allowed us to determine the suitability of the land on which each cemetery was located. Table 2 presents the results of the empirical equations for each cemetery evaluated.

**Table 2:** Validation of empirical equations for determining the suitability of territory in the setting of cemeteries.

CEMETERIES	VALIDATION OF EQUATIONS WITH FIELD VARIABLES								
	Eq 1	Eq 2	Eq 3	Eq 4	Eq 5	Eq 6	Eq 7	Eq 8	Eq 9
Number of variables	10	9	8	7	6	5	4	3	2
Lumbisí	3,3	3,3	3,2	3,3	3,3	3,3	3,4	3,5	3,7
Chillogallo - La Libertad	3,3	3,3	3,3	3,3	3,4	3,4	3,4	3,5	3,3
Tambillo	2,8	2,7	2,7	2,7	2,6	2,6	2,7	2,6	2,3
Aloasí	2,7	2,6	2,6	2,6	2,5	2,5	2,4	2,5	2,3
Nanegal	3,5	3,5	3,5	3,5	3,4	3,4	3,4	3,4	3,0
Descanso eterno	3,2	3,2	3,2	3,2	3,1	3,1	3,1	3,1	3,0
Yaruquí	2,6	2,6	2,5	2,5	2,5	2,5	2,4	2,5	2,3
Puellaro	2,2	2,2	2,1	2,0	1,9	1,8	1,6	1,5	1,3
Chavezpamba	2,8	2,8	2,7	2,7	2,7	2,6	2,5	2,5	2,3
Uyumbicho	3,1	3,1	3,1	3,2	3,2	3,2	3,3	3,5	3,7
Guangopolo	2,7	2,6	2,6	2,6	2,5	2,4	2,3	2,2	2,0
Nono	3,2	3,2	3,2	3,2	3,2	3,1	3,0	3,1	3,0
Tababela	3,2	3,2	3,2	3,2	3,3	3,3	3,3	3,3	3,3
Tumbaco	2,8	2,8	2,7	2,7	2,6	2,5	2,4	2,5	2,3
Calderón	2,5	2,4	2,3	2,3	2,3	2,2	2,2	2,2	2,0

The validation of the empirical equations proposed in Ref. [12] is essential to ensure the reliability and applicability of the mathematical model in the description and prediction of the exposed problem, thereby providing a foundation for decision-making. As mentioned above, the established features define the suitability of the land based on the study variables, which are cited in the order of relevance. Therefore, according to the bibliography, a cemetery may be classified as critical or unsuitable if, upon incorporating the data of its variables in the equations of this study, the result ranges between 4.01 and 5.00, indicating a greater probability of environmental contamination. Otherwise, it was evaluated as slightly adequate, moderately adequate, very suitable, and completely adequate, according to the aforementioned ranges [12]. Upon execution of the macro of the spreadsheet application containing 135 empirical equations, the cemeteries were categorized into three groups: those that aligned with the initial theoretical category assigned in the literature [12], those that had a higher or lower category than the original studies, and those that did not correspond with the original studies.

Cemeteries that corresponded with the original categories were Lumbisí, Yaruquí, Uyumbicho, Guangopolo, and Tumbaco, maintaining the same categorization as that initially presented [12], irrespective of the number of variables considered. These cemeteries maintained their categories in the theoretical analysis and in the run with variables measured in the field and updated the bibliography in different equations (Eqs. (1), (2), (3), (4), (5), (6), (7), (8), (9))). For example, Lumbisí in the original studies was slightly adequate, and when values were determined in the field and were obtained from an updated bibliography, the category remained unchanged.

The cemeteries that were different from the original categories were Chillogallo-La Libertad, Aloasí, Nanegal, Chavezpamba, Calderón, and Tababela because they presented a degree of difference in their original suitability, associated with the variables utilized. They were updated by field measurements or bibliography, which resulted in changes to their categorization. For example, Chillogallo-La Libertad, initially classified as unsuitable, was reclassified as slightly suitable following the update; specifically, owing to the criticality of its location, it was decreased by one category.

Finally, cemeteries that did not align with the original categories were Tambillo, Descanso Eterno, Puéllaro, and Nono. They had a variation greater than that of a single category, which is beyond scope of simple data adjustment. We reviewed the literature used in the initial run and observed errors in the use of theoretical data, such as slope and distance to water bodies. In this study, the data for all the variables used were measured in the field or from an updated bibliography; therefore, the level of reliability increased.

Puéllaro was identified as a special case in work performed in the field. The sandy loam soil characteristics did not provide water table data. The only value presented corresponded to a sample for which nearby vegetation was evaluated as retaining moisture, which might be associated with the results presented; therefore, the value used in the water table was assigned to be zero. Thus, to verify the approximation of the results when using the empirical equations, an analysis of the three cemeteries cited in existing research [12], Nanegal, Tumbaco, and Calderón, was used as a reference.

The results obtained in the development of the validation of empirical equations for determining the suitability of the territory for the subsequent location of cemeteries demonstrated that, for 10, eight, and six variables, Nanegal was a “slightly suitable” area. Tumbaco was also in a “slightly adequate” zone, and Calderón was in a “moderately adequate” zone (Table 3). Similarly, we observed that as the number of variables increased, the results gained precision in the resulting accuracy of the equations.

**Table 3:** Determination of suitability coefficients of territory in location of cemeteries, compared with those of [12].

Sites	Present study				Crisanto-Perrazo et al. [12]			
	Number of variables			Category	Number of variables			Category
	10	8	6		10	8	6	
Nanegal	3,3	3,3	3,2	Slig. Adeq.	4,05	4,11	4,18	Not Adeq.
Tumbaco	2,9	2,8	2,7	Mod. Adeq.	2,63	2,49	2,39	Mod. Adeq.
Calderón	2,3	2,2	2,2	Mod. Adeq.	1,92	1,8	1,75	Very Adeq.

**Note:** Comparison of results in the validation of empirical equations to determine the validity of the equations.

By demonstrating the small difference between categorizations, the reliability of the empirical equations was validated for the subsequent adaptation of the equations in future territorial plans for cemetery construction. Traffic lights were used to classify the results into five categories. A red result indicates a high probability of contamination, while a dark green result indicates a low probability of contamination. Eqs. (1), (2), (3), (4), (5), (6), (7), (8), (9)) were also validated for other cemeteries located outside the study area, such as Asia Cañete in Peru, Shenzhen Longshan in China, Palmira in Colombia, and Nuestro Padre Jesús de Murcia in Spain. Thirty-six equations were used, and the results are presented in Table 4.

**Table 4:** Validation of empirical equations for determining suitability of the territory in the setting of international cemeteries.

CEMETERIES	VALIDATION OF EQUATIONS								
	Eq 1	Eq 2	Eq 3	Eq 4	Eq 5	Eq 6	Eq 7	Eq 8	Eq 9
Asia Cañete	3,8	3,8	4,0	4,0	4,0	4,1	4,1	4,2	4,0
Shenzhen Longshan Permanent	2,4	2,3	2,2	2,1	2,0	1,9	1,9	1,8	1,3
Palmira	2,4	2,3	2,4	2,4	2,3	2,2	2,3	2,4	2,3
Nuestro Padre Jesús	2,2	2,2	2,1	2,1	2,0	1,9	2,0	2,1	2,3

**Note:** The results were obtained through an Excel spreadsheet, programmed with the empirical equations.

As shown in Table 4, Shenzhen Longshan and Nuestro Padre Jesús were at suitability levels of two and three, respectively. Therefore, these two cemeteries are in a territory suitable for the desired objective. However, Asia Cañete was at suitability levels of four (slightly suitable) and five (unsuitable). Therefore, collecting field data is highly recommended to validate suitability before issuing a municipal construction permit. Palmira had a suitability level of three (moderately adequate) and did not vary with the number of considered variables.

## 4 Discussion

The use of empirical equations is a quick, efficient alternative to the decision-making processes. The present evaluation was related to the assessment of land suitability for cemetery construction compared to the impact level on the environmental pollution of its surroundings. Each equation was developed with different numbers of variables for the potential case if the available information was incomplete. However, the most severe weighting was for groundwater level (m), distance to water sources (m), maximum annual precipitation (mm), and slope (%). Therefore, the equations with the highest precision were Eqs. (6), (7), (8), (9)). The majority of the variables necessitated experimental determination because of insufficient information on the official pages of the corresponding countries, which is a limitation of this study.

According to the information collected during the development of this experimental research, AM 3523 of the Ecuadorian MSP and Decree 2262/1974 of the Spanish Ministry of the Interior established that a favorable scenario for the setting of a cemetery is dry soil [29], with clay or clay loam characteristics [65,66] and low precipitation levels within an area of shallow slopes. Failure to comply with these parameters would potentially accelerate or decelerate the decomposition process of corpses, particularly in the presence of close geological faults in the area [70,71]. Furthermore, the groundwater levels must fulfill a minimum depth of 2.5 m and a minimum distance of 200 m from drinking water sources [59]. Additionally, the range of favorable temperatures was established to be 21–38 °C, because putrefaction is facilitated by high temperatures [69].

The 136 equations executed in the study area were consistent and useful, responding to the manipulation in terms of the number of variables used, and facilitating a solid conclusion regarding their application. When the same equations were used in Latin America, Asia, and Europe, their consistency was maintained, demonstrating their independence from geospatial changes. The 36 equations used to evaluate the suitability of the sites worked well and had the same consistency across scenarios, irrespective of whether two or 10 variables were used. From a practical perspective, these tools are convenient and cost-effective for decision-making processes involving high economic expenditure, which may include health, environmental, and social aspects.

Based on the results, priority should be assigned to establish the risk level acceptance that the territorial authority may need to assume when granting operating permits for final disposal undertakings. The criteria established by Icontec may also have been adopted in the Colombian Technical Guide [94]. Therefore, the presented equations demonstrate their functionality, regardless of the latitude at which they are applied. This finding may serve as a conclusive rationale for their potential as a universally used tool for locating final disposal sites, thereby saving resources for the corresponding decision-makers and authorities.

## 5 Conclusions

This study validated the empirical equations for determining the suitability of a territory for setting cemeteries. The validity and reliability of the mathematical relationships were verified by using 136 equations. The results demonstrate the validity of the equations and the necessity for data measured in the field and/or updated bibliography. Notably, the precision of the equations decreased when the number of analyzed variables decreased.

After running the equations, the values were obtained for categories classified as completely, very, and moderately suitable. In these territories, the necessary permits can be obtained for their operation and use as cemeteries. However, if the analysis demonstrates that the territory is not suitable or is slightly suitable, the recommendation is not to issue operating permits and/or measure variables in the field.

The developed equations are feasible for both the pre- and post-construction stages of a cemetery. In both cases, the most important variables (water table, distance to water sources, precipitation, slope, and soil type) can be considered before construction, avoiding future contamination problems. They can also be applied in the post-construction stage as corrective measures, because they consider variables such as cemetery age, number of graves, and population density, which influence the probability of environmental contamination.

Therefore, these equations can be easily used as quick, economical tools to define the construction settings of cemeteries worldwide, allowing them to be part of the regulations and standards in local or regional territorial planning plans.

## CRediT authorship contribution statement

**Miguel Falconí-Vélez:** Writing – original draft, Software, Investigation, Funding acquisition, Formal analysis, Data curation. **Tania Crisanto-Perrazo:** Writing – review & editing, Supervision, Resources, Project administration, Methodology, Investigation, Funding acquisition, Formal analysis, Data curation, Conceptualization. **Wendy Cachaguay Viracucha:** Resources, Investigation, Funding acquisition, Formal analysis, Data curation. **Silvana Suntaxi Pachacama:** Resources, Investigation, Funding acquisition, Formal analysis, Data curation. **Jonathan Guayasamín-Vergara:** Resources, Methodology, Investigation, Funding acquisition, Formal analysis, Data curation. **Arianna García-Bazurto:** Investigation, Formal analysis, Data curation. **Theofilos Toulkeridis:** Writing – review & editing, Writing – original draft, Resources, Methodology, Investigation, Funding acquisition, Formal analysis.

## Declaration of competing interest

The authors declare that they have no known competing financial interests or personal relationships that could have appeared to influence the work reported in this paper.
